# Supplementary material for: A dimensionless framework for predicting submarine fan morphology
Source: Nat Commun. 2022 Dec 8;13:7563. doi: 10.1038/s41467-022-34455-7 (PMC9732052; doi:10.1038/s41467-022-34455-7)
Supplement: Supplementary file 1 — Supplementary Information [file 41467_2022_34455_MOESM1_ESM.pdf]

Supplementary information for 'A dimensionless framework for predicting submarine fan morphology'

By Abdul Wahab, David C. Hoyal, Mrugesh Shringarpure, and Kyle M. Straub

Case ID	Fr_D[1] [1/1]	p[2] [1/1]	Slope [degree]	U[3] [m/s]	u_*[4] [m/s]	D_{50}[5] [um]	Dmax[6] [um]	Sorting [1/1]	Ws[7] [m/s]	Duration[8] [hour]
1	0.46	0.032	0.065	0.9	0.06	24	463	1.8	0.0019	30210
3	0.63	0.032	0.136	1.3	0.08	25	645	2.0	0.0025	22088
6	1.00	0.032	0.460	2.0	0.13	23	1178	2.4	0.0041	13809
7	1.17	0.032	0.750	2.4	0.15	22	1497	2.6	0.0048	11807
9	1.60	0.032	2.080	3.2	0.20	17	2632	3.1	0.0065	8633
11	0.46	0.055	0.065	0.9	0.06	45	463	1.4	0.0032	30210
21	0.46	0.078	0.065	0.9	0.06	65	463	1.2	0.0050	30210
26	1.00	0.078	0.460	2.0	0.13	81	1178	1.7	0.0100	13809
31	0.46	0.101	0.065	0.9	0.06	83	463	1.1	0.0059	30210
33	0.63	0.101	0.136	1.3	0.08	94	645	1.2	0.0081	22088
36	1.00	0.101	0.460	2.0	0.13	109	1178	1.5	0.0129	13809
37	1.17	0.101	0.750	2.4	0.15	113	1497	1.6	0.0151	11807
39	1.60	0.101	2.080	3.2	0.20	114	2632	1.9	0.0206	8633
51	0.46	0.148	0.065	0.9	0.06	115	463	0.9	0.0086	30210
53	0.63	0.148	0.136	1.3	0.08	135	645	1.0	0.0117	22088
56	1.00	0.148	0.460	2.0	0.13	165	1178	1.2	0.0188	13809
57	1.17	0.148	0.750	2.4	0.15	175	1497	1.3	0.0220	11807
59	1.60	0.148	2.080	3.2	0.20	188	2632	1.6	0.0300	8633
71	0.46	0.194	0.065	0.9	0.06	145	463	0.7	0.0113	30210
73	0.63	0.194	0.136	1.3	0.08	172	645	0.8	0.0154	22088
76	1.00	0.194	0.460	2.0	0.13	219	1178	1.0	0.0247	13809
77	1.17	0.194	0.750	2.4	0.15	235	1497	1.1	0.0288	11807
79	1.60	0.194	2.080	3.2	0.20	265	2632	1.4	0.0394	8633
87	1.17	0.21	0.750	2.4	0.15	265	1497	1.1	0.0323	11807
88	1.37	0.21	1.232	2.75	0.17	284	1953	1.2	0.0377	12096
91	0.46	0.240	0.065	0.9	0.06	173	463	0.6	0.0140	30210
93	0.63	0.240	0.136	1.3	0.08	207	645	0.7	0.0191	22088
96	1.00	0.240	0.460	2.0	0.13	270	1178	0.9	0.0305	13809
97	1.17	0.240	0.750	2.4	0.15	294	1497	1.0	0.0357	11807
99	1.60	0.240	2.080	3.2	0.20	341	2632	1.3	0.0488	8633

[1]Densimetric Froude number; [2]Rouse number; [3]Mean inlet velocity; [4]Mean inlet shear velocity; [5]Mean sand diameter; [6]Maximum sand diameter; [7]Mean inlet settling velocity of suspended material; [8]Total simulation time on a super computer

Supplementary Table 1| Select model variables used in this study.

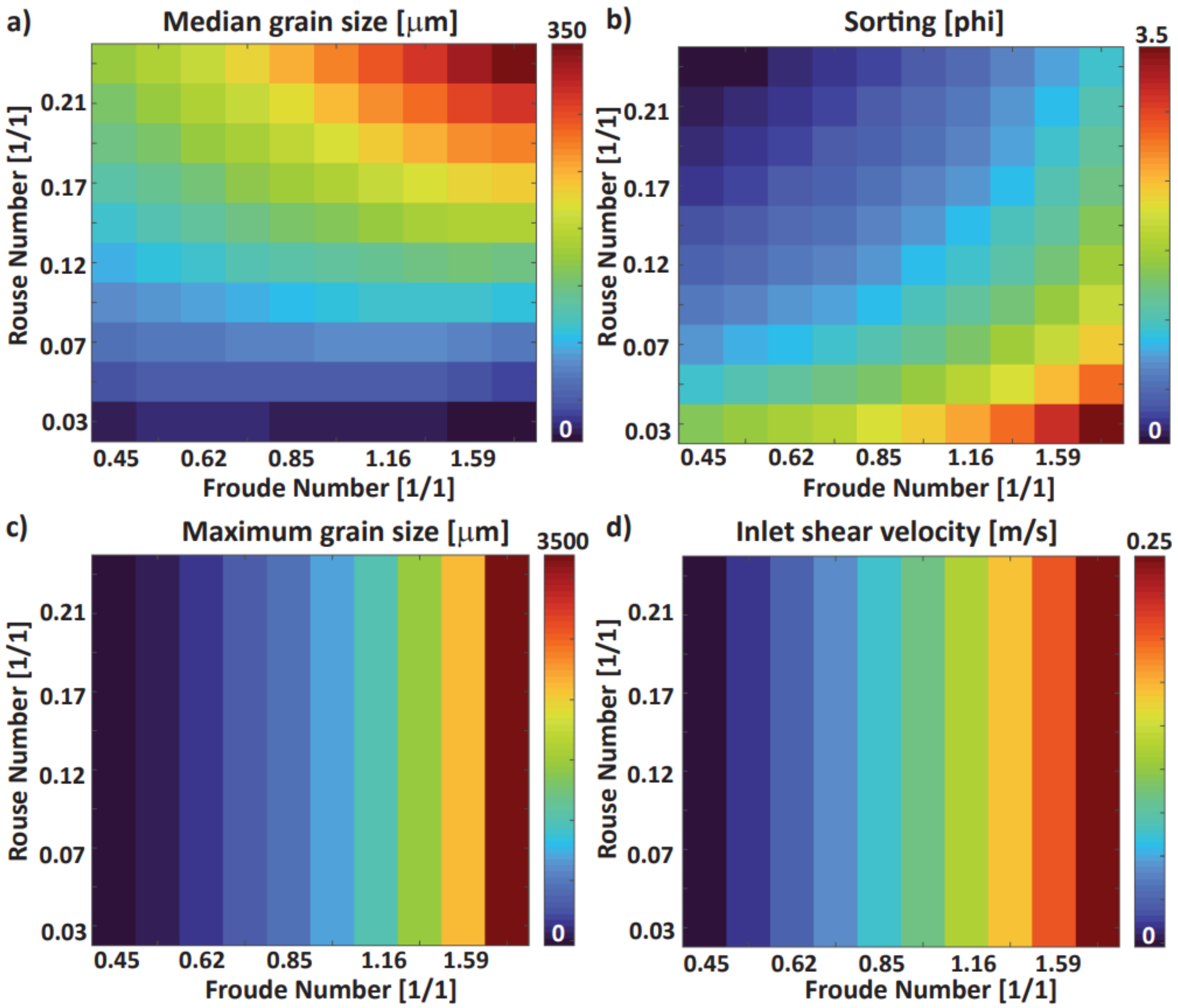

Supplementary Figure 1| Select parameters design for the dimensionless regime. a, Median grain sizes, **b.** Sorting, **c,** Maximum grain size**, d,** Inlet shear velocity.

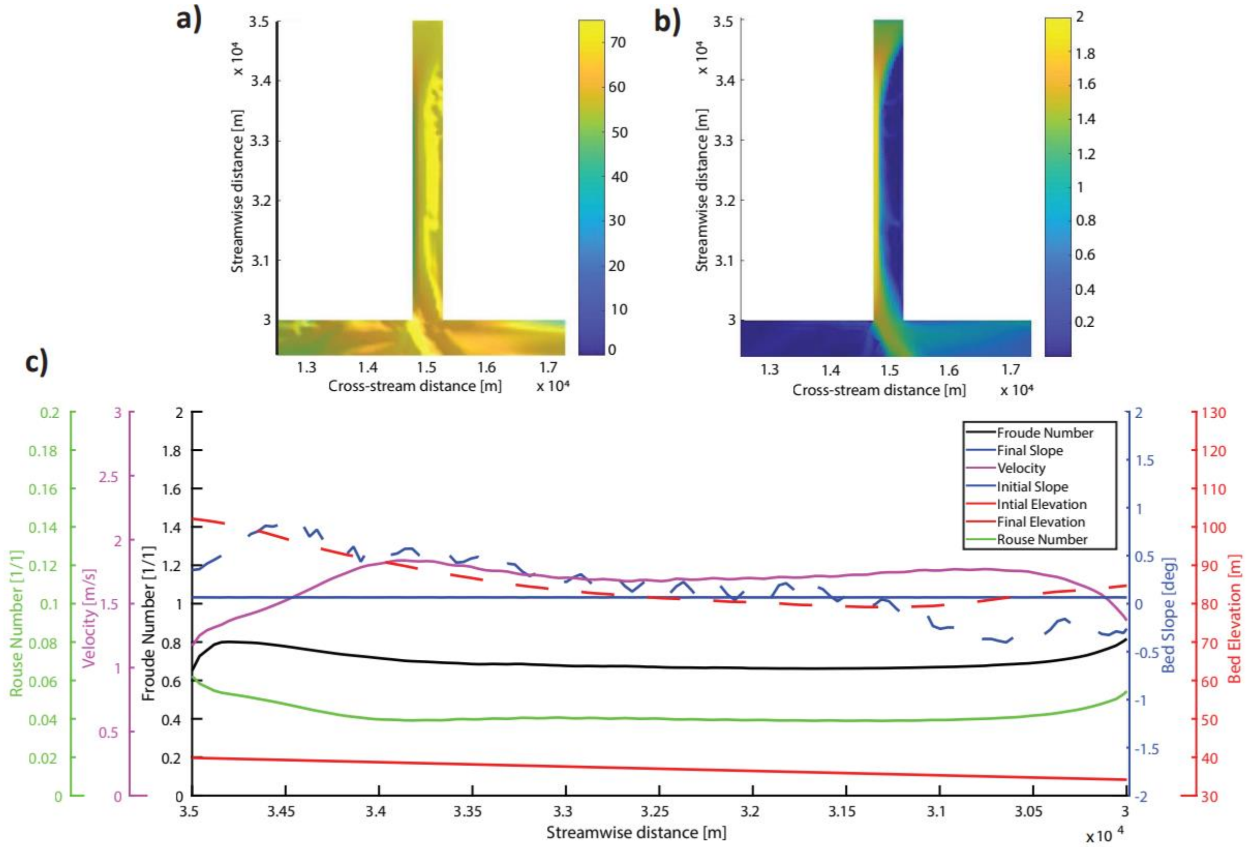

Supplementary Figure 2| Streamwise trends in flow hydraulics and bed surface properties within the inlet channel at the end of a Subcritical (Fr_D < 1) and low *p* simulation. a-b, Maps show deposit thickness and velocity magnitude within the active inlet channel. The inlet channel is 5km in streamwise and 500 m in cross-stream length. Flow starts at the inlet at 3.5 x 10^4 m. **c,** 1D plots of bed surface elevations, bed slopes, velocity magnitude, Rouse and Froude numbers are extracted from the active inlet channel shown in a&b. Further information on boundary conditions for this simulation are displayed adjacent to case ID 21 in supplementary Table 1.

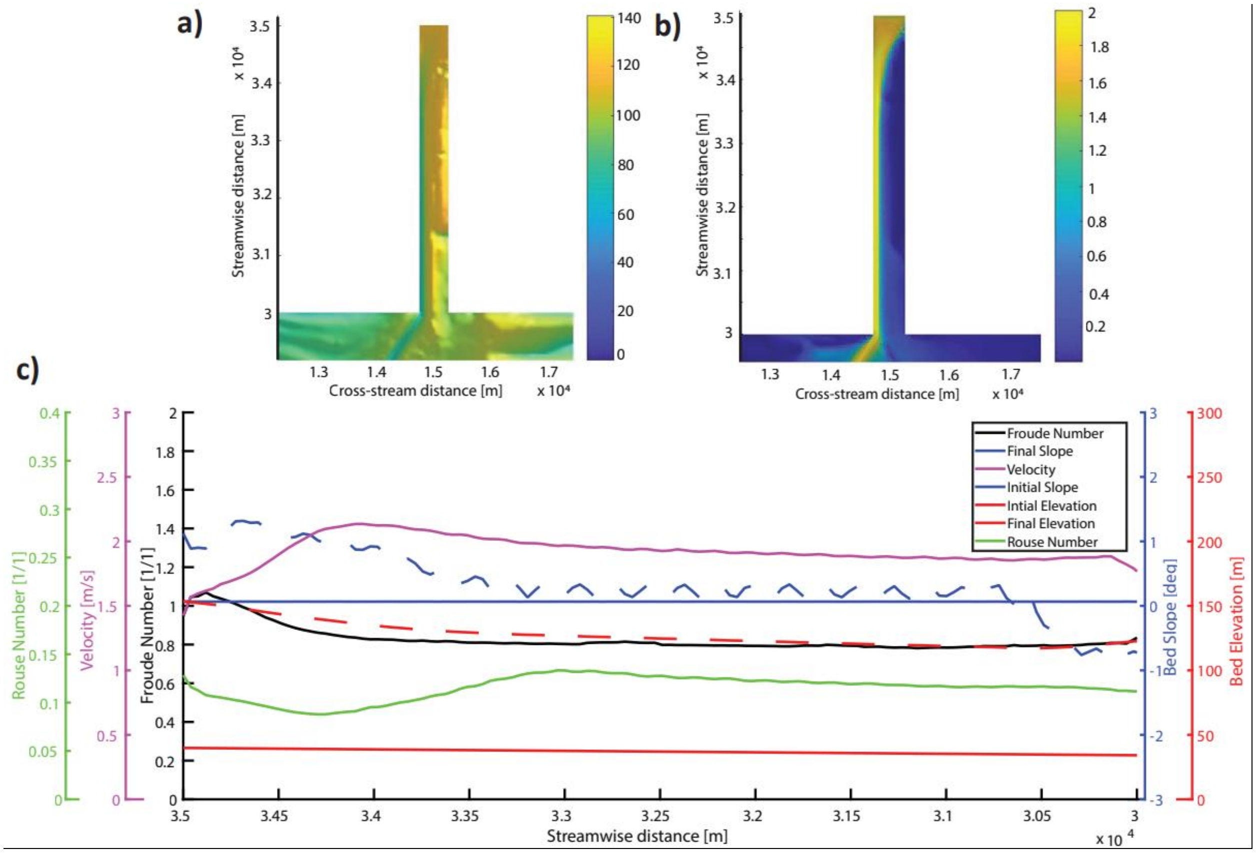

Supplementary Figure 3| Streamwise trends in flow hydraulics and bed surface properties within the inlet channel at the end of a Subcritical (Fr_D < 1) and high *p* simulation. a-b, Maps show deposit thickness and velocity magnitude within the active inlet channel. The inlet channel is 5km in streamwise and 500 m in cross-stream length. Flow starts at the inlet at 3.5 x 10^4 m. **c,** 1D plots of bed surface elevations, bed slopes, velocity magnitude, Rouse and Froude numbers are extracted from the active inlet channel shown in a&b. Further information on boundary conditions for this simulation are displayed adjacent to case ID 71 in supplementary Table 1.

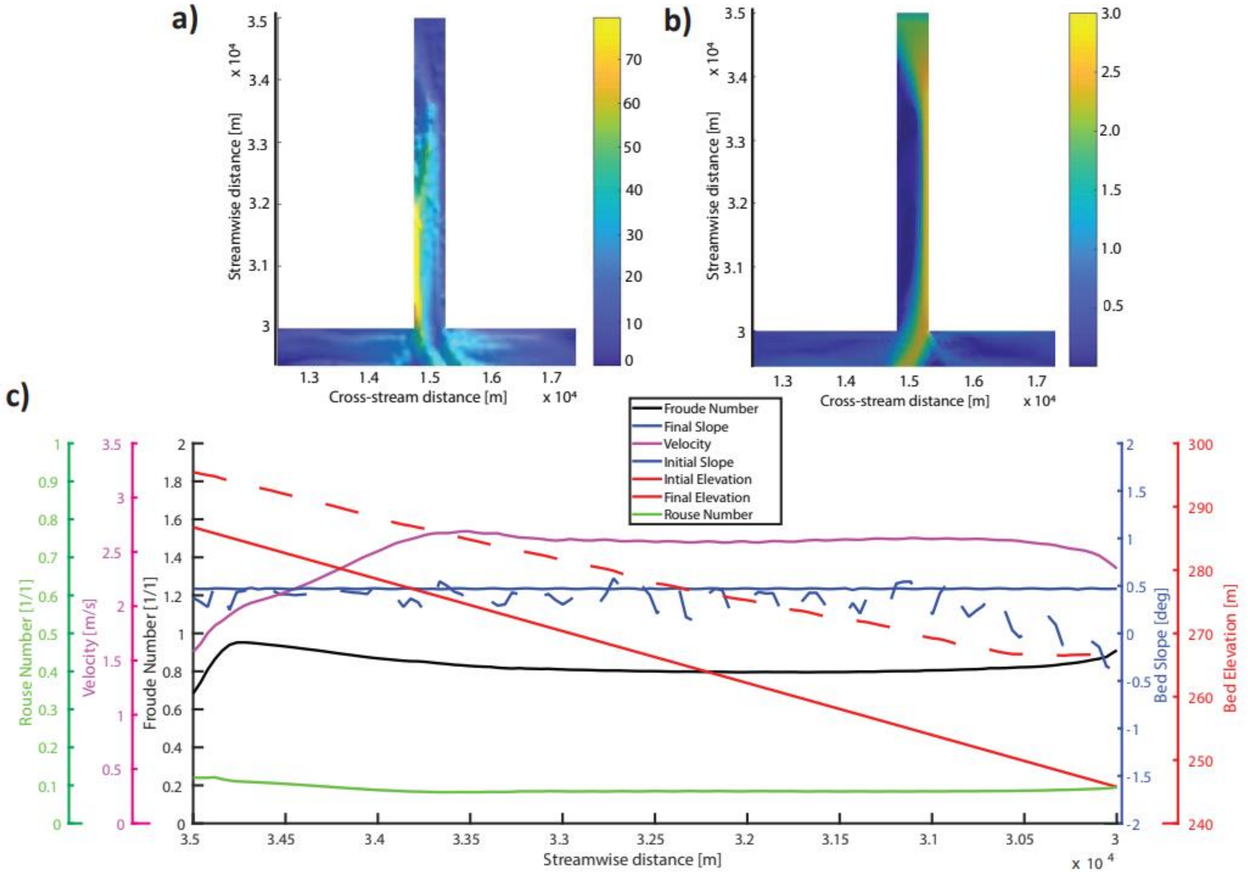

Supplementary Figure 4| Streamwise trends in flow hydraulics and bed surface properties within the inlet channel at the end of a Transcritical (Fr_D ~ 1) and low *p* simulation. a-b, Maps show deposit thickness and velocity magnitude within the active inlet channel. The inlet channel is 5km in streamwise and 500 m in cross-stream length. Flow starts at the inlet at 3.5 x 10^4 m. **c,** 1D plots of bed surface elevations, bed slopes, velocity magnitude, Rouse and Froude numbers are extracted from the active inlet channel shown in a&b. Further information on boundary conditions for this simulation are displayed adjacent to case ID 26 in supplementary Table 1.

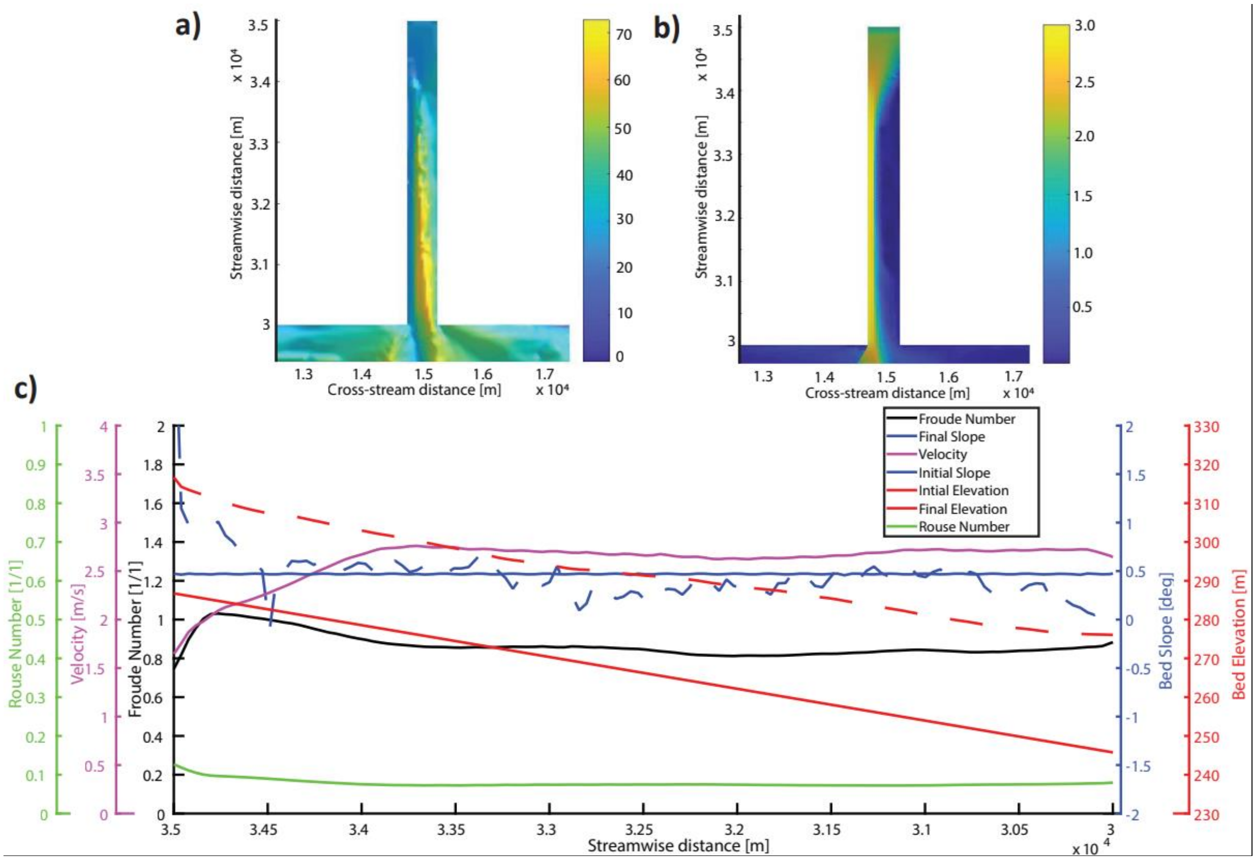

Supplementary Figure 5| Streamwise trends in flow hydraulics and bed surface properties within the inlet channel at the end of a Transcritical (Fr_D ~ 1) and relatively high p simulation. a-b, Maps show deposit thickness and velocity magnitude within the active inlet channel. The inlet channel is 5km in streamwise and 500 m in cross-stream length. Flow starts at the inlet at 3.5 x 10^4 m. **c,** 1D plots of bed surface elevations, bed slopes, velocity magnitude, Rouse and Froude numbers are extracted from the active inlet channel shown in a&b. Further information on boundary conditions for this simulation are displayed adjacent to case ID 36 in supplementary Table 1.

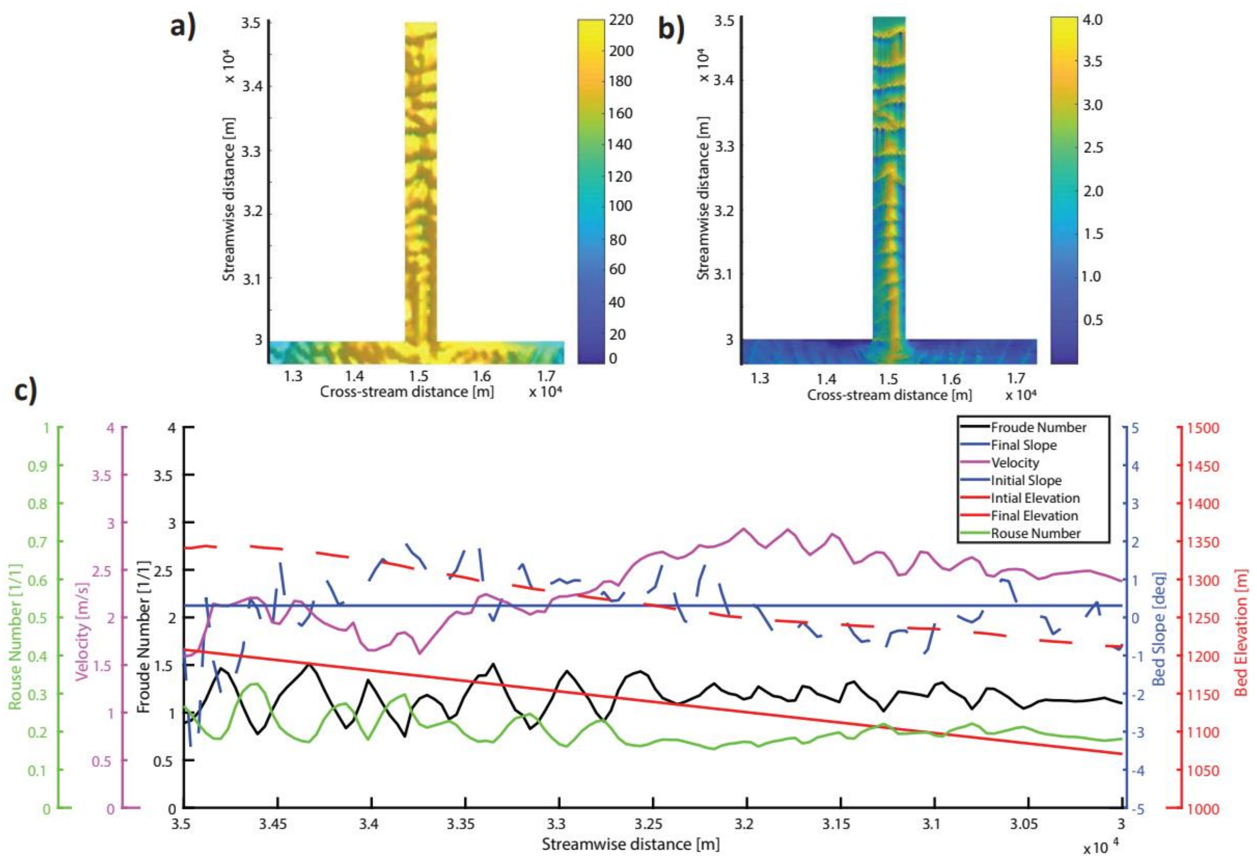

Supplementary Figure 6| Streamwise trends in flow hydraulics and bed surface properties within the inlet channel at the end of a Supercritical ($Fr_D > 1$) simulation. **a-b,** Maps show deposit thickness and velocity magnitude within the active inlet channel. The inlet channel is 5km in streamwise and 500 m in cross-stream length. Flow starts at the inlet at 3.5 x 10^4 m. **c,** 1D plots of bed surface elevations, bed slopes, velocity magnitude, Rouse and Froude numbers are extracted from the active inlet channel shown in a&b. Further information on boundary conditions for this simulation are displayed adjacent to case ID 99 in supplementary Table 1.

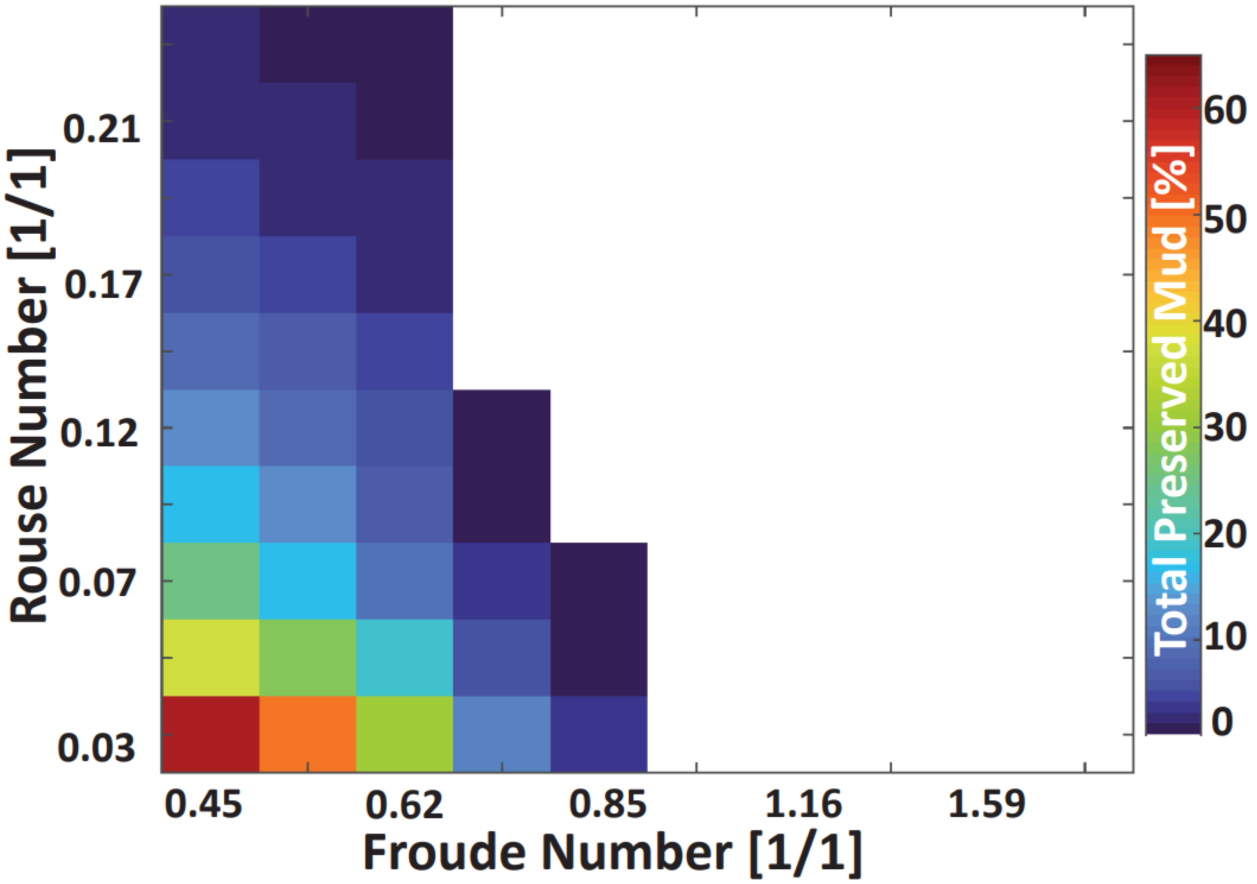

Supplementary Figure 7| Preservation of mud across the dimensionless regime. Matrix shows preservation of total mud content in all simulated submarine fans. The white region denotes models that are predominantly sandy and did not deposit any significant mud content.

Supplementary Note 1

We utilize thickness maps to measure rugosity of the simulated submarine fans. The maps are interpolated onto a Cartesian grid from raw data that is stored initially on a triangular irregular network (TIN). The grid transformation from TIN to a Cartesian grid is achieved using standard and publically available algorithms. We provide public access to four model runs that cover different corners of the regime diagram and contains subcritical, transcritical, and supercritical simulations. We subsample the number of thickness maps due to size limitations as an average model output data exceeds 1 terabyte. The processed maps are stored as 3D arrays that are structured in space (x-location) by space (y-location) by time (elevations). We also provide a Matlab script along with thickness data that can measure rugosity of the simulated submarine fans.
