# Supplementary material for: A dimensionless framework for predicting submarine fan morphology
Source: Nat Commun. 2022 Dec 8;13:7563. doi: 10.1038/s41467-022-34455-7 (PMC9732052; doi:10.1038/s41467-022-34455-7)
Supplement: Supplementary file 3 — Description of Additional Supplementary Files [file 41467_2022_34455_MOESM3_ESM.pdf]

Description of Additional Supplementary Files

File Name: Supplementary Movie 1
Description: **Subcritical submarine fan simulation with continuous flow.** Movie shows evolution of cumulative deposit thickness, velocity magnitude (top surface), Froude Number (top surface), and median grain sizes (top surface) for a select subcritical case with continuous flow. Additional forcing conditions for this select model run are highlighted adjacent to case id 11 in Supplementary Table 1. The subcritical fan in this movie is characterized by a distributive transport pattern, sinuous channels that switches between one to several active channels, a hierarchy of bifurcations, avulsions and lateral channel mobility, and an overall mud rich deposit.

File Name: Supplementary Movie 2
Description: **Transcritical submarine fan simulation with continuous flow.** Movie shows evolution of cumulative deposit thickness, velocity magnitude (top surface), Froude Number (top surface), and median grain sizes (top surface) for a select transcritical case with continuous flow. Additional forcing conditions for this select model run are highlighted adjacent to case id 36 in Supplementary Table 1. The transcritical fan in this movie is characterized by a single low-sinuosity channel that progrades into the basin, formation of cyclic steps, avulsions, and a deposit that preserves both sand and mud.

File Name: Supplementary Movie 3
Description: **Supercritical submarine fan simulation with continuous flow.** Movie shows evolution of cumulative deposit thickness, velocity magnitude (top surface), Froude Number (top surface), and median grain sizes (top surface) for a select transcritical case with continuous flow. Additional forcing conditions for this select model run are highlighted adjacent to case id 87 in Supplementary Table 1. The supercritical fan in this movie is characterized by a short low-sinuosity channel that undergoes avulsions and backstepping, formation of cyclic steps, and a sand rich deposit.

File Name: Supplementary Movie 4
Description: **Subcritical submarine fan simulation with intermittent flow.** Movie shows evolution of cumulative deposit thickness and velocity magnitude (top surface) for a select subcritical case with intermittent flow. Additional forcing conditions for this select model run are highlighted adjacent to case id 21 in Supplementary Table 1, expect for the total duration of run time (36264 hours) to account for time periods of no flow. The subcritical fan in this movie exhibits similar overall morphodynamic behavior to the fan in Supplementary movie 1.

File Name: Supplementary Movie 5
Description: **Transcritical submarine fan simulation with intermittent flow.** Movie shows evolution of cumulative deposit thickness and velocity magnitude (top surface) for a select transcritical case with intermittent flow. Additional forcing conditions for this select model run are highlighted adjacent to case id 36 in Supplementary Table 1, expect for the total duration of run time (16512 hours) to account for time periods of no flow. The transcritical fan in this movie exhibits similar overall morphodynamic behavior to the fan in Supplementary movie 2.

File Name: Supplementary Movie 6

Description: **Supercritical submarine fan simulation with intermittent flow**. Movie shows evolution of cumulative deposit thickness and velocity magnitude (top surface) for a select supercritical case with intermittent flow. Additional forcing conditions for this select model run are highlighted adjacent to case id 88 in Supplementary Table 1, expect for the total duration of run time (12096 hours) to account for time periods of no flow. The supercritical fan in this movie exhibits similar overall morphodynamic behavior to the fan in Supplementary movie 3.
